# Effect of yeast culture supplementation in sows during late gestation and lactation on growth performance, antioxidant properties, and intestinal microorganisms of offspring weaned piglets

**DOI:** 10.3389/fmicb.2022.1105888

**Published:** 2023-01-13

**Authors:** Yalei Liu, Xinlin Jia, Junlei Chang, Xuemei Jiang, Lianqiang Che, Yan Lin, Yong Zhuo, Bin Feng, Zhengfeng Fang, Jian Li, Lun Hua, Jianping Wang, Zhihua Ren, Mengmeng Sun, De Wu, Shengyu Xu

**Affiliations:** ^1^Animal Disease-Resistance Nutrition, Ministry of Education, Ministry of Agriculture and Rural Affairs, Key Laboratory of Sichuan Province, Animal Nutrition Institute, Sichuan Agricultural University, Chengdu, Sichuan, China; ^2^Sichuan Province Key Laboratory of Animal Disease and Human Health, Key Laboratory of Environmental Hazard and Human Health of Sichuan Province, College of Veterinary Medicine, Sichuan Agricultural University, Chengdu, China; ^3^College of Science, Sichuan Agricultural University, Ya'an, China

**Keywords:** yeast cultures, sows, weaned piglets, growth performance, antioxidant properties, intestinal microorganisms

## Abstract

**Introduction:**

The effects of maternal addition of yeast cultures on offspring gut development and intestinal microorganisms are not yet known, so the aim of this study was to investigate the effects of maternal addition of yeast cultures to the diet of sows during late gestation and lactation on growth performance, antioxidant properties and intestinal microorganisms of offspring weaned piglets.

**Methods:**

40 Landrace × Yorkshire sows (3–7 of parity) with similar backfat were randomly divided into two treatment groups: control diet (CON) and control diet +2.0 g/kg yeast culture (XPC), and the trial started on day 90 of gestation and ended on day 21 of lactation.

**Results:**

The results showed that maternal addition of yeast culture significantly increased weaned piglet weight and mean daily gain (*p* < 0.05), with a tendency to increase litter weight gain (*p* = 0.083) and liver weight (*p* = 0.076) compared to the control group. The content of thymus malondialdehyde (MDA) was significantly higher (*p* < 0.05) and the content of colon total antioxidant capacity (T-AOC) was significantly lower (*p* < 0.05) in the offspring weaned piglets of the XPC group compared to the control group. The expression of thymus *SOD1* and *SOD2*, spleen *SOD1*, jejunum *SOD2*, and colon *GPX1, SOD1*, and *SOD2* were significantly downregulated in the XPC group of offspring weaned piglets compared with the control group (*p* < 0.05). The intestinal morphology and the content of short-chain fatty acids in colonic chyme did not differ between the two groups (*p* > 0.05). Compared with the control group, the XPC group significantly increased the relative abundance of colonic chyme Bacteroidetes (*p* < 0.05), tended to decrease the relative abundance of *Lactobacillus* (*p* = 0.078), and tended to increase the relative abundance of *Alloprevotella* (*p* = 0.055). The XPC group significantly upregulated *Blautia* and *Fournierella* (*p* < 0.05) and significantly downregulated *Candidatus_Competibacter*, *Nitrospira*, *Dechloromonas*, *Haliangium*, and *Oscillospira* (*p* < 0.05).

**Discussion:**

In conclusion, maternal addition of yeast cultures improved the growth performance of offspring weaned piglets and changed the intestinal microbial community, but did not improve their antioxidant performance.

## 1. Introduction

In order to reduce the number of non-productive days and maximize the productivity of the sow, farms use the strategy of early weaning. Early weaned piglets have an underdeveloped immune system and are exposed to stress from various stressors such as physiological (separation from the sow, underdeveloped system), psychological (mixing with other litters, facing fighting and establishing a new social hierarchy) and nutritional (change from breast milk to solid feed), which leads to delayed growth and easy diarrhea after weaning (Modina et al., 2019; Xiong et al., 2019; Upadhaya and Kim, 2021). The main manifestations are a reduction in feed intake within 24–48 h, slowed or even negative growth, and increased susceptibility to pathogens (Lallès et al., 2007; Jayaraman and Nyachoti, 2017). Studies have shown that weaning older, more mature pigs helps prevent many of the adverse gastrointestinal effects associated with weaning stress (Craig et al., 2017; Wensley et al., 2021). In addition, reduced piglet organ weights, restricted digestion of colostrum and reduced serum immunoglobulin concentrations can lead to delayed development, resulting in differences in birth weight and subsequent growth of sow offspring (Craig et al., 2019). Therefore, nutritional and management strategies can be implemented prior to weaning to provide piglets with a good stress tolerance and thus better cope with weaning stress. For nutritional strategies, in-depth research can be conducted in sow nutrition to have a long-term beneficial effect on offspring growth performance by improving maternal uterine condition and lactational breast milk condition. It has been shown that maternal nutritional interventions can affect the intestinal health and growth performance of offspring (Cao et al., 2014; Liu et al., 2016; Chen et al., 2017), so it is important to investigate the effects of maternal nutritional interventions on offspring.

Yeast cultures are a class of biological products consisting of metabolites produced during the anaerobic fermentation of Saccharomyces cerevisiae and some live yeast (Jin et al., 2017). Yeast cultures have various functions, such as maintaining the intestinal health of animals, improving production performance, improving feed nutrition, and promoting the metabolism of the body (Fan et al., 2012; Diao, 2016; Burdick Sanchez et al., 2021). Yeast cultures have been widely used in swine production in recent years, and studies have found that the addition of yeast cultures to sow diets from late gestation to lactation improves feed intake, immune status, milk production, milk quality, and fecal microbiota, thereby improving the growth performance of piglets (Zhao et al., 2022). However, the effect of maternal addition of yeast cultures on offspring gut development and gut microbiota is unknown and needs further study. We hypothesized that maternal addition of yeast cultures would improve the growth and development of offspring weaned piglets. To test this hypothesis, we investigated the effects of adding yeast cultures to sow diets during late gestation and lactation on growth performance, antioxidant properties, intestinal morphology, short-chain fatty acids, and intestinal microorganisms of offspring weaned piglets.

## 2. Materials and methods

The yeast cultures (Diamond V, United States) used in the test contained a variety of nutrients, including oligosaccharides, proteins, peptides, amino acids, yeast-derived enzymes, nucleic acids, and etc. The trial was conducted at Yile breeding pig farm (Dekang group Co., Ltd., China). All animal procedures were approved by the Animal Care and Use Committee of Sichuan Agricultural University (Ethical Approval Code: SICAU20220119).

### 2.1. Experimental design

40 Landrace × Yorkshire sows (3–7 of parity) with similar backfat (16.80 ± 0.42 mm) were randomly divided into two treatment groups: control diet (CON) and control diet +2.0 g/kg yeast culture (XPC), with 20 replicates per treatment and 1 sow per replicate. The trial started on day 90 of gestation and ended on day 21 of lactation. At the day 20 of lactation, 6 litters of piglets with similar average weight were selected from each group, weighed one by one after fasting overnight, and one piglet close to the average weight of the litter was selected from each weaned litter for slaughter, totaling 12 piglets (half of each male and half of each female).

### 2.2. Feeding management

The nutritional needs of basal diet for sows during gestation and lactation are formulated according to National Research Council (2012) (NRC, United States). The sows in the control group were fed the basal diet and the sows in the yeast culture group were supplemented with an additional 2.0 g/kg XPC on top of the basal diet. The composition and nutritional levels of the basal diet are shown in Supplementary Table S1. Gestating sows were fed twice a day (8:30; 14:30) with a total of 3 kg of basal diet. Sows are not fed on the day of farrowing, 2 kg on the following day, and then gradually increase 1 kg per day. Sows started to feed freely on day 7 of lactation, three times a day (8:30; 11:30; 17:30). During the whole test period, sows were free to drink water, pens were cleaned daily, disinfected and dewormed regularly, and kept the air circulation and temperature constant in the house.

### 2.3. Sample collection

On the day of weaning (21 days old), 10 ml of blood was collected from the anterior vena cava of piglets, blood was loaded into sodium heparin anticoagulation tubes, left at room temperature for 30 min, then centrifuged at 3500 r/min at 4°C for 15 min, and the supernatant plasma was divided and stored at −20°C for measurement. After the piglets were slaughtered, the intestinal tissues were removed, the duodenum, jejunum and ileum were separated, and about 2 cm of the middle section of each intestine was cut and fixed in 4% paraformaldehyde solution for the determination of intestinal morphology. In addition, the middle segments of duodenum, jejunum, ileum and colon were collected, and the thymus, liver, spleen and mesenteric lymph nodes were rinsed with saline and blotted dry with absorbent paper, then divided into lyophilization tubes, snap-frozen in liquid nitrogen and stored at −80°C for measurement (Mou et al., 2018). The colonic chyme was divided into lyophilization tubes and then snap frozen in liquid nitrogen and stored at −80°C for testing (Wan et al., 2020).

### 2.4. Growth performance

After sow farrowing, record the average daily feed intake (ADFI) of sow, born alive, litter weight of born alive and average alive piglet birth weigh. After weaning, record the number of offspring weaned piglets, weaned piglet weight, weaned litter weight, calculate the average daily weight gain and litter weight gain.

### 2.5. Intestinal morphology

Morphological samples of duodenum, jejunum and ileum were sent to Yibaidao Technology Co., Ltd. (Chengdu, China) for determination. The samples were processed for paraffin preparation for HE staining, and Image Pro Plus 6.0 (media cybernetics, Inc., Rockville, MD, United States) software was applied to measure 5 villus height (VH) as well as corresponding crypt depth (CD) per field, making a total of 10 villi and their corresponding crypts. The V/C ratio is equal to the VH value divided by the CD value.

### 2.6. Antioxidant index

Sample pretreatment: All tissues were ground into powder using a mortar containing liquid nitrogen, and then weighed about 0.1 g of tissue powder and added saline in the ratio of powder weight: saline volume = 1:9, homogenized in an ice bath using a homogenizer, and then centrifuged at 4,000 r/min for 10 min at 4°C (Mou et al., 2020). The supernatant was divided and then used in a BCA protein concentration determination kit (Beyotime Biotechnology, Shanghai, China) to determine the protein concentration of the homogenate.

Catalase (CAT), glutathione peroxidase (GSH-Px), malondialdehyde (MDA), superoxide dismutase (SOD), and total antioxidant capacity (T-AOC) were measured in plasma and tissues using the corresponding kits (Grace Biotechnology Co., Ltd., Suzhou, China), and all indexes were measured according to the instructions (Bradford, 1976).

### 2.7. Gene expression

The expression of antioxidant-related genes *GPX1* (glutathione peroxidase1), *SOD1* (superoxide dismutase1), *SOD2* (superoxide dismutase2), and *CAT* (catalase) in weaned piglet tissues was determined by quantitative real-time fluorescence PCR (RT-PCR). Tissue RNA was extracted using RNAiso Plus (TaKaRa, Japan), the concentration and quality of RNA was determined using a UV spectrophotometer (NanoDrop 2000, Thermo, United States), and the integrity of RNA was detected using gel electrophoresis. Using HiScript ® III RT SuperMix for qPCR (+gDNA wiper) kit (Vazyme, R323, Nanjing, China) removes genomic DNA from the sample under the reaction condition of 42°C for 2 min. Then a reverse transcription reaction system was prepared for reverse transcription to obtain cDNA at 37°C for 15 min; 85°C, 5 s. Fluorescent quantitative PCR was performed using the ChamQ Universal SYBR qPCR Master Mix quantification kit (Vazyme, Q711, Nanjing, China) according to the instructions. Statistical results were obtained using *β-actin* as an internal reference gene and the 2^−∆∆Ct^ method was used to calculate the relative expression of target genes (Livak and Schmittgen, 2001). The target gene primer sequences (primers were synthesized by Tsingke Biotechnology Co., Ltd., Beijing, China) are shown in Supplementary Table S2.

### 2.8. Colonic chyme SCFAs and microbial flora

The colonic chyme was mixed with ultrapure water and the supernatant was added with metaphosphoric acid and crotonic acid, mixed and the supernatant was added with chromatographic methanol, mixed and the supernatant was filtered through a 0.22 μm filter membrane, and then the content of short-chain fatty acids (SCFAs) was determined by gas chromatography (Varian, GC CP3800). In addition, the colonic chyme was sent to Novogene Technology Co., Ltd. (Beijing, China) for 16 s rRNA sequencing. The general steps are as follows: DNA extraction and detection of samples, PCR amplification, product purification, library preparation and library inspection, NovaSeq up-sequencing, splicing and filtering of the raw data obtained from sequencing, and noise reduction by DADA2 to obtain the final Amplicon Sequence Variants (ASVs) and feature tables. Subsequently, the obtained ASVs were compared with the database using the classify-sklearn module in the QIIME2 software to obtain species information for each ASV. For the obtained ASVs, abundance, Alpha diversity calculation, and Venn diagram were analyzed, and in addition, the differences between different samples (groups) were found from them by Beta diversity index inter-group variance analysis, and Principal Co-ordinates Analysis (PCoA; Xu et al., 2020; Li et al., 2021). *T*-test statistical analysis was selected to test the significance of differences in species composition and community structure of the samples.

### 2.9. Statistical analysis

IBM SPSS Statistics 27 software was used for independent sample T test. Before statistical analysis, Shapiro Wilk and Levene’s tests were used to test the normality and homogeneity of variance of all data. If the data did not conform to the normal distribution, nonparametric tests were used. Results are expressed as “mean ± standard error,” with *p* < 0.05 considered as significant difference and 0.05 ≤ *p* < 0.1 considered as significant trend.

## 3. Results

### 3.1. Growth performance

Compared with the control group, maternal addition of XPC significantly increased offspring weaning piglet weight (*p* < 0.05) and significantly increased average daily weight gain of offspring weaned piglets (*p* < 0.05), with a tendency to increase sow ADFI (*p* = 0.081) and piglet litter gain (*p* = 0.083). However, there was no significant effect on the number of live births, number of offspring weaned piglets, litter weight of born alive, average alive piglet birth weigh, and weaned litter weight (*p* > 0.05; Table 1).

**Table 1:** Effects of maternal supplementation with XPC on growth performance of offspring weaned piglets.

Items	CON	XPC	*P*-value
Born alive	15.15 ± 0.72	14.70 ± 0.70	0.656
Number of weaned piglets	11.8 ± 0.34	11.75 ± 0.33	0.916
Litter weight of born alive, kg	21.54 ± 0.82	20.91 ± 0.95	0.622
Average alive piglet birth weigh, kg	1.44 ± 0.03	1.43 ± 0.03	0.871
Weaned piglet weight, kg	5.72 ± 0.19^b^	6.31 ± 0.18^a^	0.030
Weaning litter weight, kg	67.39 ± 3.00	73.65 ± 2.24	0.103
Average daily gain, kg	0.20 ± 0.01^b^	0.23 ± 0.01^a^	0.022
Litter gain, kg	45.85 ± 3.08	52.74 ± 2.33	0.083
Sow ADFI	5.78 ± 0.20	6.27 ± 0.19	0.081

Data are expressed as mean ± standard error. Different lowercase letters in the same row indicate significant differences (*p* < 0.05). ADFI, average daily feed intake of sows during lactation. CON, sows fed basal diet; XPC, sow fed basal diet + 2.0 g/kg XPC.

### 3.2. Organ index

Compared with the control group, maternal addition of XPC significantly increased slaughter weight of offspring weaned piglets (*p* < 0.05) and tended to increase liver weight (*p* = 0.076), but had no significant effect on spleen weight and organ index of offspring weaned piglets (*p* > 0.05; Table 2).

**Table 2:** The effect of maternal supplementation with XPC on the organ index of offspring weaned piglets.

Items	CON	XPC	*P*-value
**Weight (kg)**			
Slaughter weight	5.76 ± 0.07^b^	6.30 ± 0.14^a^	0.002
Liver	0.14 ± 0.01	0.17 ± 0.01	0.076
Spleen	0.01 ± 0.00	0.01 ± 0.00	0.311
**Organ index (%)**			
Liver index	2.45 ± 0.18	2.53 ± 0.16	0.749
Spleen index	0.19 ± 0.01	0.18 ± 0.01	0.434

Data are expressed as mean ± standard error. Different lowercase letters in the same row indicate significant differences (*p* < 0.05). *n* = 6. CON, sows fed basal diet; XPC, sow fed basal diet + 2.0 g/kg XPC.

### 3.3. Intestinal morphology

The results showed that there was no significant difference in the VH, CD, V/C ratio and number of goblet cells in the duodenum, jejunum and ileum in the XPC group compared with the control group (*p* > 0.05; Supplementary Table S3).

### 3.4. Antioxidant properties

The content of thymic MDA was significantly higher and the content of colonic T-AOC was significantly lower in the offspring weaned piglets of the XPC group compared to the control group (*p* < 0.05), while there was no significant difference in the antioxidant indexes of other tissues between the two groups (*p* > 0.05; Tables 3, 4).

**Table 3:** Effects of maternal supplementation with XPC on plasma and tissue antioxidant properties of offspring weaned piglets.

Items	CON	XPC	*P*-value
**Plasma**			
CAT, U/ml	162.78 ± 22.02	128.16 ± 11.40	0.193
GSH-Px, U/ml	318.15 ± 14.82	312.43 ± 8.54	0.745
MDA, nmoL/ml	1.54 ± 0.21	1.53 ± 0.12	0.974
SOD, U/ml	22.50 ± 2.46	24.93 ± 1.22	0.429
T-AOC, μmoL Trolox/ml	1.17 ± 0.02	1.16 ± 0.01	0.887
**Liver**			
CAT, U/mg prot	669.52 ± 43.26	749.72 ± 51.14	0.259
GSH-Px, U/mg prot	20.59 ± 1.62	15.81 ± 1.37	0.055
MDA, nmoL/mg prot	2.44 ± 0.43	2.22 ± 0.41	0.716
SOD, U/mg prot	2.79 ± 0.31	2.23 ± 0.19	0.157
T-AOC, nmoL Trolox/mg prot	73.74 ± 4.41	74.24 ± 1.46	0.918
**Thymus**			
CAT, U/ml	151.46 ± 6.33	127.07 ± 11.10	0.105
GSH-Px, U/ml	11.09 ± 0.64	12.69 ± 1.31	0.306
MDA, nmoL/ml	0.55 ± 0.14^b^	1.11 ± 0.17^a^	0.029
SOD, U/ml	8.85 ± 0.46	8.42 ± 0.36	0.475
T-AOC, nmoL Trolox/ml	136.92 ± 2.73	136.69 ± 8.47	0.980
**Spleen**			
CAT, U/mg prot	128.96 ± 8.31	110.45 ± 7.02	0.120
GSH-Px, U/mg prot	20.92 ± 1.63	19.56 ± 0.85	0.475
MDA, nmoL/mg prot	1.73 ± 0.56	1.26 ± 0.18	0.452
SOD, U/mg prot	7.55 ± 0.25	7.05 ± 0.31	0.109
T-AOC, nmoL Trolox/mg prot	120.25 ± 9.00	109.81 ± 1.90	0.873
Lymph nodes			
CAT, U/mg prot	33.68 ± 2.80	29.33 ± 2.87	0.303
GSH-Px, U/mg prot	2.63 ± 0.35	3.19 ± 0.71	0.494
MDA, nmoL/mg prot	1.12 ± 0.22	2.07 ± 0.60	0.167
SOD, U/mg prot	6.27 ± 0.06	5.87 ± 0.35	0.458
T-AOC, nmoL Trolox/mg prot	71.33 ± 2.33	66.19 ± 2.04	0.139

Data are expressed as mean ± standard error. Different lowercase letters in the same row indicate significant differences (*P* < 0.05). *n* = 6. CON, sows fed basal diet; XPC, sow fed basal diet + 2.0 g/kg XPC. CAT, catalase; GSH-Px, glutathione peroxidase; MDA, malondialdehyde; SOD, superoxide dismutase; T-AOC, total antioxidant capacity.

**Table 4:** Effect of maternal supplementation with XPC on intestinal antioxidant properties of offspring weaned piglets.

Items	CON	XPC	*P*-value
**Duodenum**			
CAT, U/ml	99.26 ± 10.40	97.25 ± 15.23	0.915
GSH-Px, U/ml	6.45 ± 1.00	5.53 ± 0.36	0.406
MDA, nmoL/ml	0.71 ± 0.20	0.73 ± 0.11	0.337
SOD, U/ml	10.59 ± 1.41	10.31 ± 0.91	0.871
T-AOC, nmoL Trolox/ml	74.69 ± 3.19	73.84 ± 2.31	0.833
**Jejunum**			
CAT, U/mg prot	69.16 ± 7.57	68.80 ± 6.38	0.971
GSH-Px, U/mg prot	2.50 ± 0.35	2.76 ± 0.28	0.579
MDA, nmoL/mg prot	0.80 ± 0.29	0.94 ± 0.25	0.712
SOD, U/mg prot	8.37 ± 0.74	7.16 ± 0.76	0.282
T-AOC, nmoL Trolox/mg prot	68.56 ± 2.50	65.02 ± 1.46	0.249
**Ileum**			
CAT, U/mg prot	20.24 ± 1.65	17.86 ± 2.12	0.465
GSH-Px, U/mg prot	1.73 ± 0.17	0.95 ± 0.30	0.058
MDA, nmoL/mg prot	1.46 ± 0.30	1.08 ± 0.65	0.362
SOD, U/mg prot	8.27 ± 0.50	7.72 ± 0.63	0.509
T-AOC, nmoL Trolox/mg prot	77.30 ± 2.88	68.15 ± 3.04	0.054
**Colon**			
CAT, U/mg prot	27.15 ± 3.49	33.14 ± 1.50	0.147
GSH-Px, U/mg prot	4.61 ± 1.29	4.27 ± 0.68	0.817
MDA, nmoL/mg prot	0.33 ± 0.09	0.24 ± 0.05	0.439
SOD, U/mg prot	6.31 ± 0.52	6.51 ± 0.67	0.818
T-AOC, nmoL Trolox/mg prot	98.77 ± 2.81^a^	85.36 ± 1.48^b^	0.026

Data are expressed as mean ± standard error. Different lowercase letters in the same row indicate significant differences (*P* < 0.05). *n* = 6. CON, sows fed basal diet; XPC, sow fed basal diet + 2.0 g/kg XPC. CAT, catalase; GSH-Px, glutathione peroxidase; MDA, malondialdehyde; SOD, superoxide dismutase; T-AOC, total antioxidant capacity.

### 3.5. Antioxidant-related gene expression

**Figure 1:**
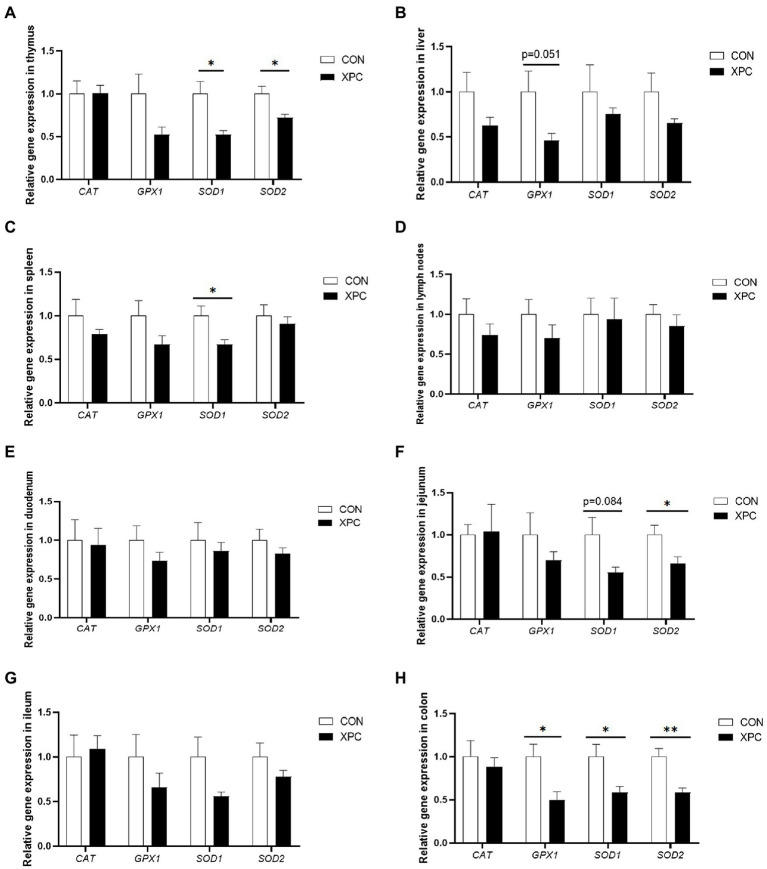
Effect of maternal supplementation with XPC on the expression of antioxidant-related genes in different tissues **(A)** thymus, **(B)** liver, **(C)** spleen, **(D)** lymph nodes, **(E)** duodenum, **(F)** jejunum, **(G)** ileum, **(H)** colon, of offspring weaned piglets. Data are expressed as mean ± standard error, *n* = 6. *p < 0.05, **p < 0.01. CON, sows fed basal diet; XPC, sow fed basal diet +2.0 g/kg XPC. *GPX*, glutathione peroxidase; *SOD*, superoxide dismutase; *CAT*, catalase.

Compared with the control group, the expression of thymus *SOD1* and *SOD2* was significantly downregulated in the XPC group of offspring weaned piglets (*p* < 0.05, Figure 1A), the expression of liver *GPX1* tended to be downregulated (*p* = 0.051, Figure 1B), and the expression of spleen *SOD1* was significantly downregulated (*p* < 0.05, Figure 1C). There was no significant difference in the expression of lymph nodes and duodenum between the two groups (*p* < 0.05, Figures 1D,E). The expression of jejunal *SOD2* was significantly downregulated (*p* < 0.05), the expression of *SOD1* tended to be downregulated (*p* = 0.081, Figure 1F). There was no significant difference in the expression of ileum between the two groups (*p* < 0.05, Figure 1G). The expression of colonic *GPX1*, *SOD1*, and *SOD2* was significantly downregulated (*p* < 0.05, Figure 1H).

### 3.6. Colonic chyme SCFAs

Maternal addition of XPC did not have a significant effect on colonic chyme SCFAs in offspring weaned piglets (*p* > 0.05; Supplementary Table S4).

### 3.7. Microbial composition of colonic chyme

**Figure 2:**
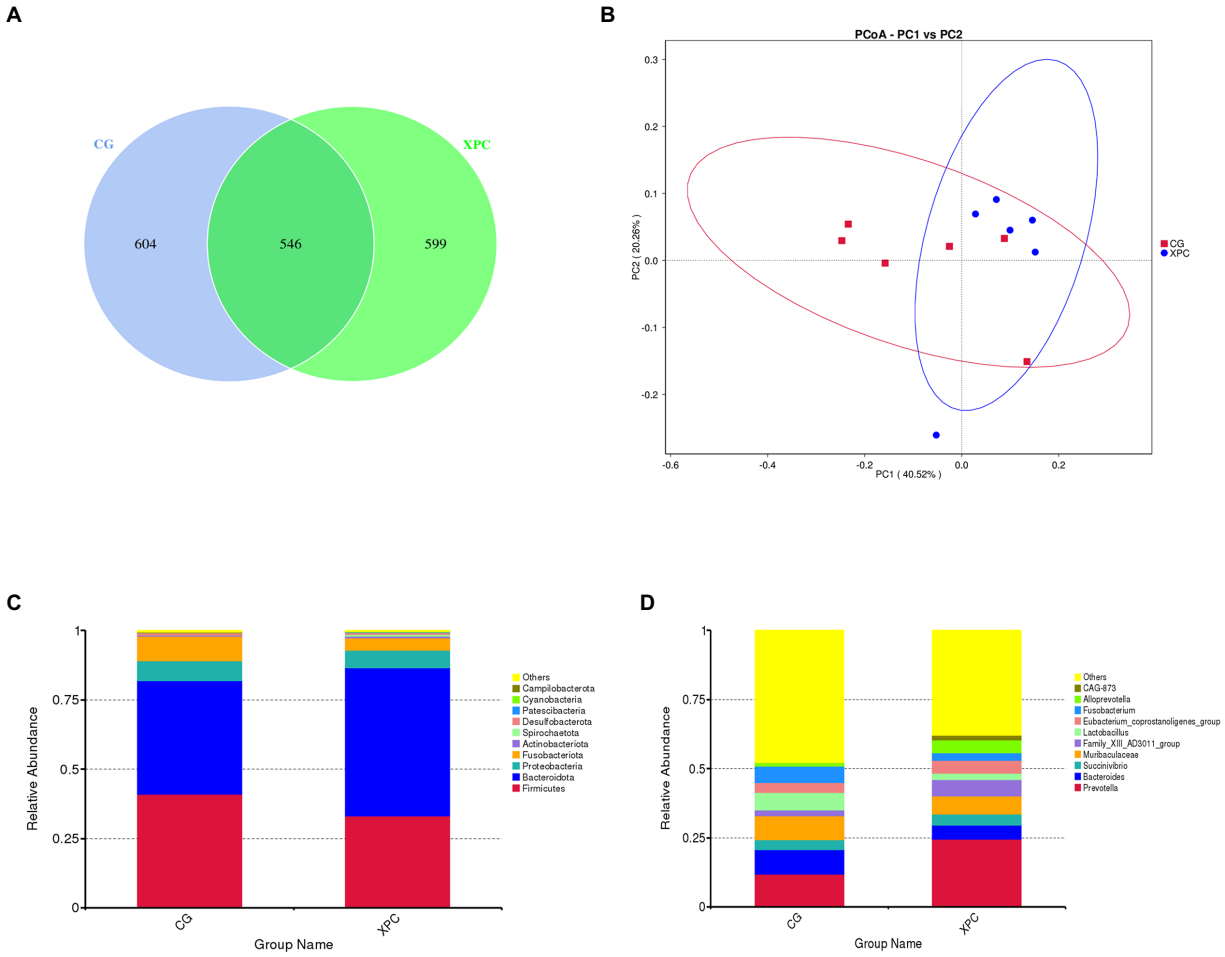
Effects of maternal supplementation with XPC on colonic microbial composition and relative abundance in offspring weaned piglets. **(A)** Amplicon Sequence Variants (ASVs) analysis. **(B)** Principal Co-ordinates Analysis (PCoA). **(C)** Relative abundance of phylum level. **(D)** Relative abundance of genus level. CG: control group (CON, sows fed basal diet); XPC, yeast culture group (sow fed basal diet +2.0 g/kg XPC). *n* = 6.

Maternal addition of XPC had no significant effect on the colonic microbial α-diversity index in offspring weaned piglets compared to the control group (*p* > 0.05; Supplementary Table S5). Both groups enjoyed 546 ASVs together, 604 and 599 ASVs specific to the control and XPC groups, respectively (Figure 2A). The analysis of the principal coordinates revealed that the colonic microbial communities of the two groups were separately aggregated and had significantly different community structures (Figure 2B).

### 3.8. Relative abundance of colonic chyme microorganisms

Firmicutes and Bacteroidetes were the dominant phylum among colonic microorganisms in offspring weaned piglets. Compared to the control group, the XPC group significantly increased the relative abundance of Bacteroidetes (*p* < 0.05; Table 5; Figure 2C), tended to decrease the relative abundance of *Lactobacillus* (*p* = 0.078), and tended to increase the relative abundance of *Alloprevotella* (*p* = 0.055; Table 6; Figure 2D).

**Table 5:** Effect of maternal supplementation with XPC on the relative abundance of colonic microbial phylum level of offspring weaned piglets (%).

Items	CON	XPC	*P*-value
Firmicutes	41.06 ± 7.88	33.09 ± 4.53	0.401
Bacteroidota	40.92 ± 5.14^b^	53.56 ± 2.06^a^	0.046
Proteobacteria	7.07 ± 2.87	6.29 ± 3.52	0.420
Fusobacteriota	8.89 ± 2.61	4.42 ± 3.04	0.262
Actinobacteriota	0.41 ± 0.15	0.64 ± 0.42	0.423
Spirochaetota	0.08 ± 0.06	0.52 ± 0.41	0.806
Desulfobacterota	0.85 ± 0.23	0.72 ± 0.24	0.721
Patescibacteria	0.06 ± 0.04	0.28 ± 0.18	0.142
Cyanobacteria	0.00 ± 0.00	0.12 ± 0.09	0.153
Campilobacterota	0.09 ± 0.05	0.10 ± 0.10	0.798
Others	0.56 ± 0.10	0.26 ± 0.12	0.078

Data are expressed as mean ± standard error. Different lowercase letters in the same row indicate significant differences (*p* < 0.05). *n* = 6. CON, sows fed basal diet; XPC, sow fed basal diet + 2.0 g/kg XPC.

**Table 6:** Effect of maternal supplementation with XPC on the relative abundance of colonic microbial genus level of offspring weaned piglets (%).

Items	CON	XPC	*P*-value
*Prevotella*	11.76 ± 5.51	24.52 ± 5.68	0.109
*Bacteroides*	8.96 ± 3.82	5.15 ± 2.42	0.262
*Succinivibrio*	3.53 ± 3.22	3.98 ± 3.89	0.149
*Muribaculaceae*	8.79 ± 2.85	6.45 ± 1.69	0.496
*Family_XIII_AD3011_group*	1.96 ± 0.72	5.97 ± 2.93	0.236
*Lactobacillus*	6.32 ± 2.51	2.30 ± 0.59	0.078
*Eubacterium_coprostanoligenes_group*	3.73 ± 0.83	4.45 ± 2.17	0.423
*Fusobacterium*	5.93 ± 2.37	2.82 ± 1.69	0.200
*Alloprevotella*	1.14 ± 0.49	4.65 ± 1.55	0.055
*CAG-873*	0.08 ± 0.01	1.67 ± 1.59	0.872
*Others*	47.79 ± 6.25	37.95 ± 1.55	0.181

Data are expressed as mean ± standard error. Different lowercase letters in the same row indicate significant differences (*p* < 0.05). *n* = 6. CON, sows fed basal diet; XPC, sow fed basal diet + 2.0 g/kg XPC.

### 3.9. Species differences in colonic chyme microorganisms

**Figure 3:**
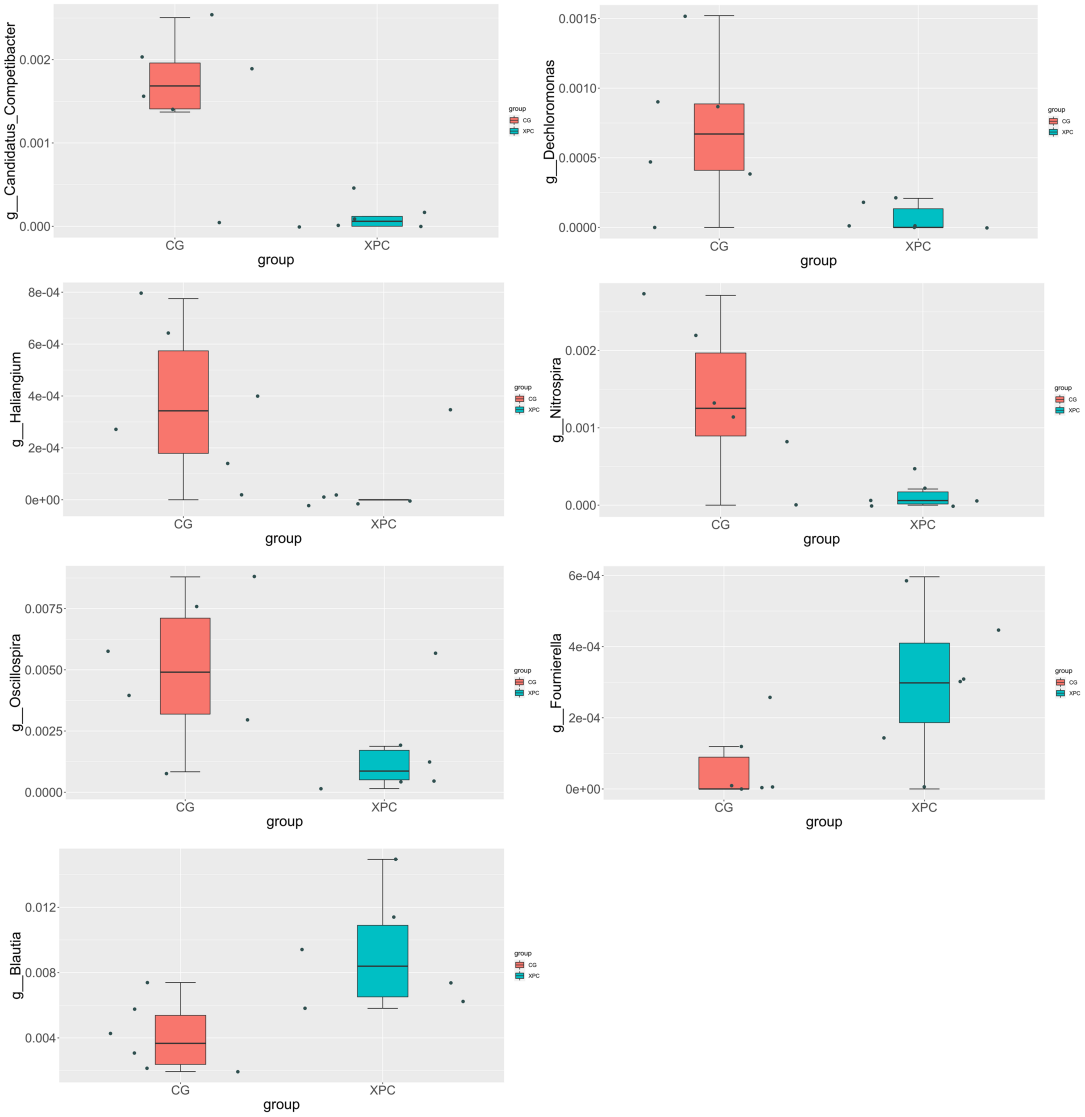
*T*-test analysis of species differences between groups. CG, control group (CON, sows fed basal diet); XPC, yeast culture group (sow fed basal diet +2.0 g/kg XPC). *n* = 6.

Compared with the control group, the XPC group significantly upregulated *Blautia* and *Fournierella* (*p* < 0.05), and significantly downregulated *Candidatus_Competibacter*, *Nitrospira*, *Dechloromonas*, *Haliangium*, and *Oscillospira* (*p* < 0.05, Figure 3).

## 4. Discussion

The development of piglets during the embryonic period and the source of nutrition such as breast milk after birth are crucial to the growth and development of piglets, so the nutritional and physiological status of the sow during the fetal and lactation stages of piglets will greatly affect the growth and development of piglets. Higher feed intake of sows during lactation increased milk production and thus contributed to higher weaning weight of piglets (Jiang et al., 2020). The results of this experiment showed that maternal addition of XPC significantly increased weaning piglet weight, average daily weight gain, and tended to increase sow ADFI and piglet litter weight gain, so we speculate that XPC is beneficial to increase the milk production of sows by increasing sow feed intake, so that piglets can get more nutrition and thus increase weaning piglet weight. It was found that the addition of brewer’s yeast fermentation products to sow diets had no significant effect on the number of live births and weaned piglets, but increased weaning litter weight and litter gain, probably due to a reduction in sow plasma urea nitrogen to improve sow protein utilization, which increased milk production without affecting colostrum and milk (Shen et al., 2011). The addition of 4% brewer’s yeast to the sow’s diet during late gestation and lactation significantly increased piglet weaning survival rate and weaning weight, significantly reduced piglet stillbirth rate, and improved sow milk production and quality (Song et al., 2017). It has also been found that the addition of yeast cultures to sows during late gestation and lactation significantly increased the average daily weight gain of piglets and improved the milk yield of sows (Zhao et al., 2022), which is consistent with the results of present experiment. Studies have shown that the nutritional status of the sow affects milk production, especially the availability of energy and protein during lactation, and that increased nutritional availability promotes mammary gland development as well as metabolism, which leads to increased milk production (Kim et al., 2000; Bass et al., 2019; Lu et al., 2019). It suggests that maternal addition of yeast cultures may increase the weaning weight of piglets as well as the average daily weight gain by increasing the milk production of the sow.

The intestine is the main digestive and absorption site of the body, in which intestinal epithelial cells play an important role. Villi cells absorb nutrients, water and electrolytes, while crypt cells secrete water and electrolytes (Xiong et al., 2019), and villi height and crypt depth are related to the number of villi and crypt cells (Hampson, 1986), and if villi become shorter and crypt deeper, which indicates a decrease in intestinal absorptive cells and an increase in secretory cells, it leads to poorer intestinal absorption and increased secretion, which may be related to piglet diarrhea (Nabuurs et al., 1993; Yang and Yu, 2021). Undigested dietary fiber is fermented by intestinal microorganisms to produce SCFAs. SCFAs play an important role in the body’s glycolipid metabolism, inflammatory response, maintenance of intestinal integrity, and immune homeostasis (Tan et al., 2014; Xu, 2017). The results of this experiment showed that maternal addition of XPC had no significant effect on intestinal morphology and colonic chyme SCFAs of offspring weaned piglets. This indicates that the intestinal digestion, absorption and secretion function of offspring could not be affected by maternal addition of XPC, and in addition, it could not affect the intestinal microbial dietary fiber fermentation of offspring. Antioxidant enzymes can scavenge reactive oxygen species (ROS) in the body and play an important role in maintaining the balance of the redox state of the body (Irshad and Chaudhuri, 2002). The results of this experiment showed that the content of thymic MDA was higher in the XPC group of offspring weaned piglets, while the content of colonic T-AOC was lower, and in addition, the expression of antioxidant-related genes in different tissues was downregulated to different degrees. It was found that the addition of selenium-enriched yeast to the diet improved the antioxidant capacity of weaned piglets (Liu, L. et al., 2021a). Another study found that the addition of yeast products to the diet increased the rate of diarrhea in weaned piglets, adversely affected both intestinal morphology and intestinal barrier, and to some extent improved the antioxidant capacity (Yang et al., 2016). Piglet diarrhea is usually associated with oxidative stress (Granot and Kohen, 2004), and the negative impact of yeast products on the piglet intestine leads to oxidative stress, which activates the antioxidant system and increases the activity of antioxidant enzymes in the body (Yang et al., 2016). Under the present conditions, XPC did not affect the intestinal morphology of the offspring weaned piglets, so it is possible that the piglets in the XPC group had lower levels of oxidants resulting in a lower redox status, or that the piglets in the XPC group had a lower antioxidant capacity themselves. It is noteworthy that piglets in the XPC group showed better growth performance, which may suggest better resistance to oxidative stress, thus facilitating growth.

Intestinal microorganisms play an important role in animal health and play an important role in nutrient metabolism, growth and development, maintenance of the intestinal barrier, immune regulation, and resistance to pathogen invasion (Yu et al., 2022). Piglets form intestinal microorganisms through contact with the sow’s birth canal, skin, feces, environmental microorganisms and by suckling milk (He et al., 2020; Zhang et al., 2022). It was found that the breast milk microbiota constitutes the initial microbiota of the newborn piglets gut and plays a crucial role in regulating newborn piglets health (Wu et al., 2006; Chen et al., 2018; Li et al., 2022). Thus, maternal addition of yeast cultures may affect the gut microbiology of piglets by influencing the composition of breast milk and sow feces microbes. This pilot study found that the relative abundance of *Alloprevotella* was higher in the XPC group and the relative abundance of *Lactobacillus* was lower compared to the control group. *Lactobacillus* as a common probiotic has good probiotic properties and significantly inhibits the multiplication of pathogenic bacteria (He et al., 2019). *Alloprevotella* is also a probiotic that mainly produces succinate and acetate, both of which have improved intestinal barrier and anti-inflammatory effects (Downes et al., 2013). Thus, while maternal addition of XPC decreased the relative abundance of the *Lactobacillus* in the intestine of offspring weaned piglets, it increased the relative abundance of another beneficial bacterium, *Alloprevotella*. Similarly, another study found that the addition of live yeast to sow diets significantly reduced the number of fecal *Lactobacillus* in piglets (Van et al., 2003), which may be related to differences in maternal transmission of XPC and the ability of XPC to better influence anti-inflammatory function in piglets.

Species difference analysis showed that *Blautia* and *Fournierella* were significantly up-regulated in the XPC group, and *Candidatus_Competibacter*, *Nitrospira*, *Dechloromonas*, *Haliangium*, and *Oscillospira* were significantly down-regulated. *Blautia* is a group of anaerobic bacteria with probiotic properties, widely present in the intestine and feces of mammals, that ferment different types of carbohydrates to produce acetic acid, lactic acid and ethanol and improve glucose metabolism in animals (Kiros et al., 2019; Liu, X. et al., 2021). *Fournierella* favors the production of short-chain fatty acids (Liu et al., 2020). Therefore, genera related to intestinal barrier, anti-inflammation and glucose metabolism were more abundant in the intestine of offspring weaned piglets in the XPC group, and these genera may have promoted the absorption of nutrients, better utilization of energy and resistance to pathogenic bacteria, ultimately promoting piglet growth and higher weaning weight. Both *Candidatus_Competibacter* and *Dechloromonas* belong to the Proteobacteria, while the Proteobacteria is associated with intestinal diseases and is mostly pathogenic and can cause inflammation (He et al., 2019). *Nitrospira* and *Haliangium* are mostly found in water bodies and soil and have a more specific function, while *Oscillospira* can produce butyric acid (Konikoff and Gophna, 2016). Therefore, XPC can increase the content of beneficial bacteria and reduce the content of harmful bacteria in the intestine of weaned piglets to some extent. It was found that the addition of yeast probiotics to the diet had no significant effect on the fecal α-diversity index of weaned piglets, but the microbiota was significantly different from the control group (Xu et al., 2018). In contrast, supplementation of live brewer’s yeast during lactation in piglets increased cecum microbial α-diversity and increased *Blautia*, *Collinsella* and *Eubacterium* (Kiros et al., 2019). The reason for the difference in test results may be the difference in yeast product, the object of addition, and the amount of additives. Another possible explanation is the difference in diet composition. The sow diet in this trial was not a corn-soybean meal diet, but a mixed diet, so the difference in sow diet largely affected the intestinal microorganisms. The addition of yeast cultures to sows may have a direct effect on breast milk as well as fecal microorganisms, and piglets’ intestinal microorganisms may also be affected through exposure to breast milk and sow feces. Therefore, a variety of factors caused the complexity of intestinal microbes in offspring weaned piglets.

## 5. Conclusion

Maternal addition of yeast cultures increased offspring piglet weaning weight, average daily weight gain and litter weight gain, and increased the content of beneficial intestinal bacteria, but had no effect on intestinal morphology and the content of SCFAs in colonic chyme, and reduced antioxidant capacity. Therefore, maternal addition of yeast cultures can improve the growth and development of the offspring to some extent, especially in terms of growth performance. Yeast cultures can be used as a potential growth promoter to promote the growth of offspring through maternal transmission.

## Data availability statement

The data presented in the study are deposited in the National Center for Biotechnology Information (NCBI) Sequence Read Archive (SRA), accession number: PRJNA914903).

## Ethics statement

The animal study was reviewed and approved by Animal Care and Use Committee of Sichuan Agricultural University.

## Author contributions

SX, YLiu, and DW designed the study. YLiu and XJia carried out the animal experiments and performed the laboratory work. YLiu, XJia, JC, XJian, LC, YLin, YZ, BF, ZF, JL, LH, JW, ZR, and MS performed the statistical analysis. YLiu wrote the paper. SX and DW revised the manuscript. All authors contributed to the article and approved the submitted version.

## Funding

This research was funded by Major Scientific and Technological Special Project of Sichuan Province (no. 2021ZDZX0009), Natural Science Foundation of Sichuan Province (2022NSFSC1628), and Sichuan Province “145” Breeding Tackle Project (2021YFYZ0008).

## Conflict of interest

The authors declare that the research was conducted in the absence of any commercial or financial relationships that could be construed as a potential conflict of interest.

## Publisher’s note

All claims expressed in this article are solely those of the authors and do not necessarily represent those of their affiliated organizations, or those of the publisher, the editors and the reviewers. Any product that may be evaluated in this article, or claim that may be made by its manufacturer, is not guaranteed or endorsed by the publisher.
