# Supplementary material for: Effect of yeast culture supplementation in sows during late gestation and lactation on growth performance, antioxidant properties, and intestinal microorganisms of offspring weaned piglets
Source: Front Microbiol. 2023 Jan 13;13:1105888. doi: 10.3389/fmicb.2022.1105888 (PMC9880171; doi:10.3389/fmicb.2022.1105888)
Supplement: Supplementary file 1 [file Table_1.docx]

**Supplementary Table 1** Composition and nutrient level of experimental basal diet

| **Items** | **Pregnancy** | **Lactation** |
| --- | --- | --- |
| **Ingredient, %** |  |  |
| Corn | 20.00 | 29.96 |
| Wheat | 13.72 | -- |
| Wheatmeal, 14%CP | 15.00 | 15.00 |
| Soybean meal, 46%CP | 6.40 | 8.70 |
| Fermented soybean meal | -- | 4.00 |
| Expanded soybean | -- | 5.00 |
| Brown rice mix | 10.00 | 10.00 |
| Sugar beet meal granulate | 6.00 | -- |
| Rice bran meal | 10.00 | 14.90 |
| Rice bran | 5.00 | 5.00 |
| Soybean skin | 7.80 | -- |
| Soybean oil | 1.55 | 2.00 |
| Calcium hydrogen phosphate type I | 1.75 | 1.31 |
| Limestone | 0.91 | 1.15 |
| Sodium chloride | 0.35 | 0.35 |
| L-Lys HCl, 98% | 0.12 | 0.22 |
| DL-Met, 98.5% | 0.05 | 0.15 |
| L-Thr, 98% | 0.02 | 0.12 |
| L-Trp, 98% | 0.02 | 0.12 |
| Sodium bicarbonate | 0.09 | -- |
| Choline chloride, 50% | 0.15 | 0.15 |
| Vitamin and mineral premix^1^ | 1.07 | 1.87 |
| Total | 100.00 | 100.00 |
| **Nutrient level^2^** |  |  |
| Digestible energy, MJ/kg | 14.38 | 14.64 |
| Crude protein, % | 13.07 | 16.22 |
| Calcium, % | 1.11 | 0.97 |
| Total phosphorus, % | 0.68 | 0.72 |
| Non-phytate phosphorus, % | 0.54 | 0.50 |

^1^Per kilogram of diet provided: Cu 20 mg; I 0.4 mg; Fe 110 mg; Mn 40 mg; Se 0.25 mg; Zn 115 mg; VA 10000 IU; VB_1_ 6.0 mg; VB_2_ 5.0 mg; VB_6_ 4.0 mg; VB_12_ 30 μg; VD_3_ 2200 IU; VE 80 IU; VK_3_ 4.0 mg; Biotin 0.3 mg; Folic acid 3.0 mg; Pantothenic acid 20 mg; Nicotinic acid 40 mg.

^2^Digestible energy, crude protein and non-phytate phosphorus are calculated values, total phosphorus and calcium are measured values.

**Supplementary Table 2** Target gene primer sequences

| **Gene** | **Primer sequence (5’→3’)** | **Accession no.** | |
| --- | --- | --- | --- |
| *GPX1* | F: GATGCCACTGCCCTCATGA | AF532927 |  |
|  | R: TCGAAGTTCCATGCGATGTC |  |  |
| *SOD1* | F: GAGCTGAAGGGAGAGAAGACAGT | NM_001190422.1 | |
|  | R: GCACTGGTACAGCCTTGTGTAT |  |  |
| *SOD2* | F: CTGGACAAATCTGAGCCCTAAC | NM_214127.2 | |
|  | R: GACGGATACAGCGGTCAACT |  |  |
| *CAT* | F: CGAAGGCGAAGGTGTTTG | NM_214301.2 | |
|  | R: AGTGTGCGATCCATATCC |  |  |
| *β-actin* | F: GGCGCCCAGCACGAT | DQ845171.1 | |
|  | R: CCGATCCACACGGAGTACTTG |  |  |

*GPX*, glutathione peroxidase; *SOD*, superoxide dismutase; *CAT*, catalase

**Supplementary Table 3** Effects of maternal supplementation with XPC on intestinal morphology and goblet cells of offspring weaned piglets

| **Items** | **CON** | **XPC** | ***P*-value** |
| --- | --- | --- | --- |
| **Duodenum** |  |  |  |
| VH, µm | 350.32 ± 16.94 | 369.58 ± 19.15 | 0.469 |
| CD, µm | 112.74 ± 9.14 | 95.54 ± 8.98 | 0.209 |
| V/C ratio | 3.26 ± 0.40 | 3.80 ± 0.05 | 0.232 |
| **Jejunum** |  |  |  |
| VH, µm | 295.60 ± 7.77 | 341.33 ± 24.82 | 0.129 |
| CD, µm | 81.79 ± 2.75 | 88.56 ± 6.55 | 0.362 |
| V/C ratio | 3.63 ± 0.15 | 3.90 ± 0.27 | 0.419 |
| **Ileum** |  |  |  |
| VH, µm | 272.78 ± 24.04 | 298.01 ± 12.57 | 0.374 |
| CD, µm | 77.01 ± 11.27 | 90.81 ± 6.94 | 0.321 |
| V/C ratio | 3.75 ± 0.43 | 3.39 ± 0.33 | 0.522 |

Data are expressed as mean ± standard error. Different lowercase letters in the same row indicate significant differences (*P* < 0.05). n = 6. CON, sows fed basal diet; XPC, sow fed basal diet + 2.0 g/kg XPC. VH, villus height; CD, crypt depth; V/C ratio, villus height/crypt depth.7

**Supplementary Table 4** Effects of maternal supplementation with XPC on SCFAs in colonic chyme of offspring weaned piglets

| **Items** | **CON** | **XPC** | ***P*-value** |
| --- | --- | --- | --- |
| Acetic acid, mmol/g | 1.35 ± 0.24 | 1.43 ± 0.14 | 0.796 |
| Propionic acid, mmol/g | 0.64 ± 0.16 | 0.67 ± 0.16 | 0.873 |
| Isobutyric acid, mmol/g | 0.07 ± 0.02 | 0.07 ± 0.02 | 0.946 |
| Butyric acid, mmol/g | 0.32 ± 0.08 | 0.35 ± 0.11 | 0.873 |
| Isovaleric acid, mmol/g | 0.20 ± 0.05 | 0.35 ± 0.16 | 0.631 |
| Valeric acid, mmol/g | 0.10 ± 0.03 | 0.09 ± 0.02 | 0.798 |
| Total acid, mmol/g | 2.68 ± 0.54 | 2.95 ± 0.44 | 0.712 |

Data are expressed as mean ± standard error. Different lowercase letters in the same row indicate significant differences (*P* < 0.05). n = 6. CON, sows fed basal diet; XPC, sow fed basal diet + 2.0 g/kg XPC.

**Supplementary Table 5** Effects of maternal supplementation with XPC on colonic microbial alpha diversity index of offspring weaned piglets

| **Items** | **CON** | **XPC** | ***P*-value** |
| --- | --- | --- | --- |
| chao1 | 371.14 ± 19.59 | 348.21 ± 21.73 | 0.451 |
| dominance | 0.07 ± 0.02 | 0.05 ± 0.01 | 0.373 |
| observed_otus | 370.67 ± 19.48 | 347.17 ± 21.25 | 0.434 |
| pielou_e | 0.68 ± 0.03 | 0.70 ± 0.02 | 0.556 |
| shannon | 5.81 ± 0.25 | 5.93 ± 0.16 | 0.712 |
| simpson | 0.93 ± 0.02 | 0.95 ± 0.01 | 0.373 |

Data are expressed as mean ± standard error. Different lowercase letters in the same row indicate significant differences (*P* < 0.05). n = 6. CON, sows fed basal diet; XPC, sow fed basal diet + 2.0 g/kg XPC.
